# The Diagnostic and Prognostic Value of Heart-Sounds

**Published:** 1871-09

**Authors:** Austin Flint


### Miscellaneous.

### The Diagnostic and Prognostic Value of Heart-Sounds.

BY AUSTIN FLINT, M. D.

It is difficult, without the lessons of clinical experience, to appreciate the fact that the intensity and quality of heart murmurs are not of much account in judging of the importance of valvular lesions. A murmur very loud, notably rough or musical, it would seem, should denote graver lesions than one which is feeble, soft, and blowing. Experience, however, shows that it is not so. A striking illustration of this fact came under my observation some time since. A gentleman from Cuba consulted me for disease of the heart. He had a musical murmur loud enough to be heard with the ear removed some inches from the chest. The sound had attracted his attention, and this first led him to see a physician. He was told that he had disease of the heart, of which he had previously had no suspicion, having no ailments referable to that organ, and, indeed, considering himself perfectly well. He became at once a medical curiosity, and he had been examined by many physicians. The case exemplified the fact that the diagnosis of a cardiac lesion is sometimes a misfortune. The man had no peace of mind after the discovery of the murmur. He relinquished his business, and came to this country for medical opinions. The lesion, as regards present importance, was innocuous; and had he remained ignorant of its existence, he would not only have been contented and comfortable, but his condition would probably have been more favorable for the preservation of health.

It follows, from what has been said, that with reference to prognosis, it is important to go further in diagnosis than to determine, from the presence of murmur, the existence of an organic disease of the heart. If we except the accident of embolism, we are warranted in saying that, as a rule, in cases of valvular lesions giving rise to murmurs, whatever be their number, intensity, and quality, there is no danger, either immediate or near at hand, so long as the heart is not enlarged; for clinical observation shows that, in general, valvular lesions cause enlargement of the heart before leading to more remote effects, which involve distress and jeopardize life. Moreover, clinical observation shows that in most cases, enlargement of the heart is produced by valvular lesions slowly, the ulterior effects being, of course, proportionately distant. I would remark, in this connection, that in order to judge of the import of organic murmurs aside from enlargement of the heart, the heart-sounds claim more attention from stethoscopists than is usually given to them. It is certain that the aortic and the pulmonary-artery second sound can generally be interrogated separately by auscultation; and I believe this statement may also be made with respect to the mitral and the tricuspid valvular element of the first sound. The absence of any abnormal modifications of these several components of the two sounds of the heart is an important point in judging of the innocuousness of valvular lesions, the existence of which is revealed by the presence of murmur.

The difference in the tolerance of chronic affections of the heart is to be considered with reference to the prognosis. What is true of most chronic diseases—namely, that the same lesions are tolerated very differently in different cases, is especially exemplified by the structural affections of the heart. It is truly astonishing how well borne, in some cases, are cardiac lesions of unusual magnitude. A case which recently came under my observation afforded a striking illustration of this fact. The patient, a man of middle age, was suffering greatly from dyspnœa in paroxysms, together with loss of appetite and general prostration, and the case ended fatally within a few weeks after the occurrence of the symptoms just named. I saw the patient a few days before his death, and found the heart enormously enlarged. The apex-beat was in the eighth intercostal space several inches without the linea mammalis; and the dullness on percussion over the præcordia was proportionately increased both in aera and degree. Here was truly a *cor bovinum.* There were present murmurs, indicating both aortic and mitral lesions. There had occurred an attack of acute articular rheumatism fifteen years ago. Now, prior to a few weeks before his death, this patient had seemed to be in excellent health, and he declared that he was so. He was a man of very active habits, engaged in business (that of a wool merchant in the country) which required much travelling. He had had on one occasion an attack of hemiplegia ofvery brief duration, which was probably attributable to embolism. With this exception he had not for many years been a patient, considering himself a healthy man. He was a man of temperate habits, but a good liver as regards diet, eating very heartily, and digesting his abundant meals without difficulty; yet it is certain that for several years there must have been very great enlargement of the heart, resulting from the valvular lesions. For some time before the occurrence of grave symptoms referable to the heart, he had done an unusual amount of mental and physical work, accompanied with much excitement; nervous æsthenia and impaired appetite ensued, and under these circumstances he began to suffer from dyspnœa. He was compelled to keep his bed; he became despondent; the existence of disease of the heart was forced upon his attention, and he failed rapidly. The history of this case represents what I have been repeatedly led to observe in other cases—to wit, the tolerance of disease of the heart, while it was advancing more or less slowly, until it had attained to a great amount, the person affected, in the meantime, not considering himself an invalid, taking no remedies, living freely, and engaged in pursuits involving activity of mind, or of body, or of both. The case alse represents a fact which I have repeatedly observed—namely, that from the time when persons with disease of the heart become patients, that is, when they become impressed with a knowledge of the existence of the disease, and are obliged to give up their usual pursuits and habits, they are apt to fail rapidly. It is a *facilis descensus* from that time. The latter fact, as well as the remarkable tolerance of the disease under the circumstances stated, teaches an instructive practical lesson.

In speaking now of the tolerance of cardiac lesions, I do not, of course, have any reference to those which have already been referred to as innocuous. I refer to lesions which are more or less serious—that is, involving either obstruction to the free passage of the blood through the orifices of the heart, or regurgitation, or both of these immediate effects combined, together with enlargement by hypertrophy or dilatation separately or in combination.

All clinical observers who have seen much of diseases of the heart must have been struck with the fact, that the inconvenience and suffering attendant on lesions the same in character and extent, differ widely in different cases.

What are the circumstances on which this variation as regards tolerance depends? The question not only has a bearing on the prognosis, but it is of great importance in relation to management. I will devote to it a few remarks.

In general terms, chronic diseases of the heart, as of other organs, are tolerated in proportion as the functions of the body, exclusive of the part diseased, are healthfully performed. The internal conditions of general health and constitutional strength relate especially to the series of functions which begin with ingestion and end with nutrition. Other things being equal, the toleration is best and longest when, *first* of all, the ingesta are ample; *second*, when digestion is active; *third*, when, owing to adequate assimilation, the constituents of the blood are in normal proportion; *fourth*, when the nutritive supplies in the blood are well appropriated; and *lastly*, when the secretory and excretory organs do their proper work. Now, a healthful performance of these functions is not incompatible with considerable damage to the central organ of the circulation; and in so far as it is practicable to maintain those functions at or near to the state of health, the toleration of disease of the heart will approximate to completeness. *Per contra*, the toleration will be incomplete in proportion as the functions of the body, exclusive of the heart, are feebly or imperfectly performed; in other words, in so far as the condition just named of general health and constitutional strength are deficient. The blood may be considered as representing the healthful performance, or otherwise, of the functions of nutritive and destructive assimilation; so that the simple phrase, *healthy blood*, comprehends the grand requirements for toleration."

In the discussion which followed Dr. Flint's paper, the following statistics, based on ninety *post mortem* examinations made in the Bellevue Hospital, New York, were brought forward by Dr. Alfred L. Loomis. They illustrate the question of how often and in what manner cardiac lesions are the direct cause of death:

It will be seen that valvular disease, cardiac hypertrophy. and dilatation, were present in fourteen cases. Of this number, heart lesions were the cause of death in seven ; death was sudden in one, and was caused by stenosis of the mitral and tricuspid orifices.

In fifteen cases valvular lesions, with cardiac hypertrophy, were present, in eleven of which the heart lesions were the cause of death ; in five of these death was sudden, and the valvular lesions were aortic in one, mitral in another, aortic and mitral in another, mitral and tricuspid in another, and mitral and pulmonic in another.

In six cases valvular lesions, with cardiac dilatation, were present. In four of these the heart lesions were the cause of death ; two died suddenly, in one the valvular lesions were mitral stenosis and aortic thickening, in the other the aortic, mitral, and tricuspid valves were all diseased.

In forty-six cases valvular lesions were present without cardiac hypertrophy or dilatation. In only two of these were heart lesions the cause of death, in neither of which was death sudden. Lesions of the coronary arteries were present in three cases; in one, death was sudden. Thrombi of the heart were present in six cases, death sudden in one.

It will also be seen that the number of deaths due directly to heart lesions was twenty-six. In nineteen cases death was sudden; number of sudden deaths due to heart lesions, ten; number of gradual deaths due to heart lesions, sixteen ; number of deaths not due to heart lesions, sixty-four. Of the nine sudden deaths not due to heart lesion, four were from cerebral apoplexy, four from uræmic convulsions, and one from croupous laryngitis.—*Exchange.* —*Richmond and Louisville Medical Journal.*

